# Lanthanide-Mediated Dephosphorylation Used for Peptide Cleavage during Solid Phase Peptide Synthesis

**DOI:** 10.3390/molecules18043894

**Published:** 2013-04-02

**Authors:** Byunghee Yoo, Mark D. Pagel

**Affiliations:** 1 MGH/MIT/HMS Athinoula A. Martinos Center for Biomedical Imaging, Massachusetts General Hospital and Harvard Medical School, Boston, MA 02129, USA; E-Mail: byunghee@nmr.mgh.harvard.edu; 2 Departments of Chemistry & Biochemistry, Biomedical Imaging and Medical Imaging, University of Arizona, Tucson, AZ 85724, USA

**Keywords:** lanthanide, dephosphorylation, peptide synthesis, solid phase

## Abstract

Lanthanide(III) ions can accelerate the hydrolysis of phosphomonoesters and phosphodiesters in neutral aqueous solution. In this paper, lanthanide-mediated dephosphorylation has been applied in aqueous media as an orthogonal cleavage condition that can be employed in conventional solid phase peptide synthesis (SPPS). A phosphorylated polymeric support for SPPS was developed using Boc chemistry. The cleavage of resin-bound phosphates was investigated with the addition of Eu(III), Yb(III), acid or base, a mixture of solvents or different temperatures. To demonstrate the utility of this approach for SPPS, a peptide sequence was synthesized on a phosphorylated polymeric support and quantitatively cleaved with lanthanide ions in neutral aqueous media. The protecting groups for side chains were retained during peptide cleavage using lanthanide ions. This new methodology provides a mild orthogonal cleavage condition of phosphoester as a linker during SPPS.

## 1. Introduction

The ligand complexes of some lanthanide(III) ions can mediate the hydrolysis of phosphomonoesters and phosphodiesters in aqueous or organic solutions [1,2,3,4,5,6]. Lanthanide(III) ions can efficiently hydrolyze phosphoric esters in neutral aqueous solution because they have a high coordination number, high ionization potential, and high substitution lability [7,8]. The high coordination number provides opportunities to simultaneously coordinate many water molecules and a phosphoric ester. The high ionization potential creates strong Lewis acidity, which lowers the pKa of a lanthanide-bound water nucleophile that can interact with the phosphoric ester. The ligand substitution lability provides an activation barrier to ligand exchange that stabilizes the lanthanide-water-phosphoric ester conformation [9,10,11,12,13]. The lanthanide-mediated catalytic hydrolysis of phosphoric esters is an active research area that has been applied to solid phase synthesis of phosphonated or phosphoamidated oligonucleotides [14,15].

Solid Phase Peptide Synthesis (SPPS) is frequently used to create peptides and peptide bioconjugates. SPPS requires selective deprotection of the amino terminus of a resin-bound peptide and selective cleavage of the peptide from the resin [16]. In addition, the side chains of amino acid residues must be deprotected, which is usually accomplished during peptide cleavage from the resin. SPPS schemes that conjugate specific side chains to a non-peptidyl ligand may require selective deprotection of some side chains before deprotecting the remaining side chains. Therefore, three orthogonal methods may be needed to deprotect and cleave peptides during some SPPS schemes.

Most SPPS methods use protecting groups and peptide-resin linkers that are acid- or base-labile. SPPS with base-labile Fmoc chemistry is arguably the most popular peptide synthesis methodology in current practice, although care must be taken with acid-base chemistry if specific side chains must be selectively deprotected. SPPS with acid-labile Boc chemistry can be performed at lower cost than Fmoc-SPPS, and is preferred for synthesizing complex peptide sequences, aggregating sequences, and base-sensitive peptide analogs. However, Boc-SPPS requires very strong acids to cleave the peptide from the resin, which limits strategies available for selective deprotection of specific side chains. To avoid these problems with acid- and base-labile chemistries, some linkers have been developed for SPPS that can be cleaved using reducing agents, UV light, ultrasonication or fluoride [17,18,19,20]. Yet the cleavage of these linkers produces organic radicals that must be rapidly scavenged before they attack the peptide product, and these cleavage methods can have difficulty achieving quantitative yields. 

To expand the choice of orthogonal deprotection and cleavage methods for SPPS, we investigated the use of a phosphoric ester as a peptide-resin linker and lanthanide(III)-mediated dephosphorylation in neutral aqueous media as a final peptide harvesting method.

## 2. Results and Discussion

### 2.1. Preparation of Phosphorylated Resins

**Scheme 1:**
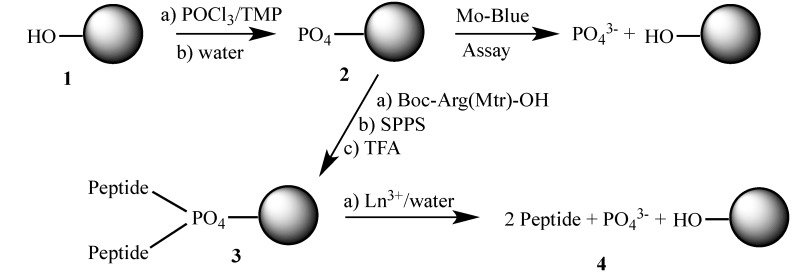
Synthesis and cleavage of a peptide using phosphate linker resin.

**Figure 1:**
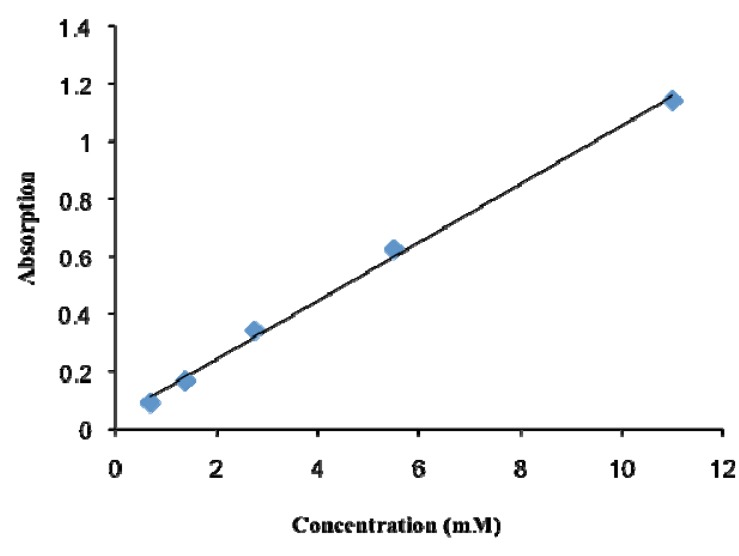
Calibration curve for phosphate quantification using KH_2_PO_4_ as a phosphate standard. The peak absorbance was observed at 825 nm, which showed a 5 nm difference. from previous reports [22]. The calibration curve showed good linearity (r^2^ = 0.997).

The general synthetic procedures are summarized in Scheme 1. A phosphorylated resin was prepared by treating a Wang resin with phosphate oxychloride. The degree of phosphorylation was quantified with a Mo-Blue assay (Figure 1), which showed an average of 35% of the hydroxyl groups of the Wang resin were phosphorylated. For comparison, two other phosphorylation methods using pyrophosphoric acid and acetylphosphate were tested too (Table 1). The phosphorylation of the Wang resin with pyrophosphoric acid formed resin-bound polyphosphates with a highly variable loading [21]. Phosphorylation with acetylphosphate ester produced only 4.5% resin loading, presumably because the hydrophobic polystyrene of the Wang resin inhibits interactions between the active acetylphosphate ester and the hydroxyl groups on the resin.

*Reagents and conditions*: Synthesis of the phosphate-loaded resin **2**: (a) Wang resin (with OH loading of 1.2 mmol/g), POCL_3_ (2 equiv.), trimethylphosphate (TMP), 60 °C, 12 h; (b) water, r.t., 2 h. Synthesis of the peptide on the solid resin **3**: (a) Wang resin (with PO_4_ loadings of 0.326 and 0.496 mmol/g), Boc-Arg(Mtr)-OH (4 equiv.), HBTU (4 equiv.), HOBt (1 equiv.), DIEA (6 equiv.), NMP, r.t., overnight; repeated for a second coupling; (b) SPPS of Z-Gly-Gly-Arg(Mtr) following standard Boc-chemistry; (c) 95% TFA, CH_2_Cl_2_, 4 h. Cleavage of the peptide and resin **4**: (a) EuCl_3_, pH 6.2, r.t., 2 h; repeated for a second cleavage.

**Table 1:** Phosphorylation of the Wang resin.

Type of Phosphate	PO_4_ loading (mmol/g)
Pyrophosphate	1.8 ± 1.4 ^1^
Acetylphosphate ester	0.054 ± 0.022
Phosphorous oxychloride	0.41 ± 0.08
Initial OH loading on resin	1.20

^1^ The PO_4_ loading that was greater than the initial OH loading on the resin indicated the formation of polyphosphates on the resin.

### 2.2. Lanthanide-Mediated Dephosphorylation

To demonstrate lanthanide-mediated dephosphorylation of the phosphorylated resin under different conditions, YbCl_3_ or EuCl_3_ solutions were added to the phosphorylated resin. These lanthanide ions were selected for this proof-of-concept study, yet future studies that investigate the utility of other rare earth ions for this chemistry application are warranted. Small (~20 mg) amounts of resin were periodically extracted from the slurry and tested for residual phosphate content using a Mo-blue assay. The phosphate cleavage was tested in distilled water, equal volumes of water and EtOH, 20% piperidine in *N*-methyl-2-pyrrolidone (NMP), and 50% trifluoroacetic acid (TFA) in CH_2_Cl_2_, and was also tested at room temperature and at 50 °C.

**Figure 2:**
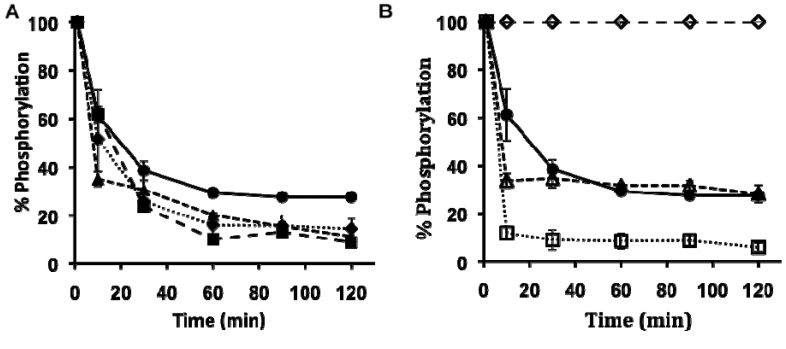
(**A**) The effect of temperature and EtOH on lanthanide-mediated hydrolysis of the phosphate groups on the phosphorylated resin. (● Eu(III) in water at pH 6.2, ▲ Eu(III) in water/EtOH, ♦ Eu(III) in water at 50 °C at pH 6.1, and ■ Eu(III) in water/EtOH at 50 °C). (**B**) The effect of lanthanides, acids and bases on hydrolysis of the phosphate groups on the phosphorylated resin. The dephosphorylations were conducted at room temperature. (◊ 50% TFA in CH_2_Cl_2_, ● Eu(III) in water at pH 6.2, △ Yb(III) in water at pH 6.3, and □ 20% piperidine in NMP). Many data points in both graphs have error bars that are smaller than the data symbol.

The water/EtOH co-solvent system improved the yield of dephosphorylation with Eu(III) at room temperature relative to only using water (Figure 2A). EtOH can promote swelling of the Wang resin that consists of hydrophobic polystyrene, which improves the diffusion of lanthanide ions into the inner matrix of the Wang resin. Therefore, using water-miscible organic solvents or hydrophilic resins may improve the dephosphorylation of a resin. Heating the resin to 50 °C also improved the yield of dephosphorylation in both solvent systems, which suggested that heat can also improve swelling of the phosphorylated Wang resin. Yb(III) showed faster initial dephosphorylation than Eu(III) (Figure 2B). Yb(III) has a smaller ionic radius and a higher charge density than Eu(III), and therefore acts as a stronger Lewis acid [23]. This leads to a faster catalytic constant for Yb(III) relative to Eu(III), which matches observations of nucleic acid dephosphorylations with lanthanide ions [24,25,26].

### 2.3. Peptide Synthesis

The phosphates were rapidly cleaved from the phosphorylated Wang resin with 20% piperidine in NMP, which demonstrated that phosphate loaded resin is not compatible with Fmoc-SPPS. The phosphate loaded resin was very stable in 50% TFA in CH_2_Cl_2_, so we investigated the utility of this resin for Boc-SPPS (Scheme 1). A model peptide sequence, CBZ-Gly-Gly-Arg(Mtr), was synthesized by double coupling Boc-Arg(Mtr)-OH to a phosphate-loaded Wang resin (200 µmol as PO_4_, 0.326 mmol per gram of resin), removing the Boc protecting group with 50% TFA in CH_2_Cl_2_, and using the same double coupling and deprotection SPPS procedure to sequentially add Boc-Gly-OH and CBZ-Gly-OH to the peptide chain. To validate the synthesis, the same SPPS sequences were repeated using a resin with greater phosphorylation (200 µmol as PO_4_, 0.496 mmol per gram of resin).

**Figure 3:**
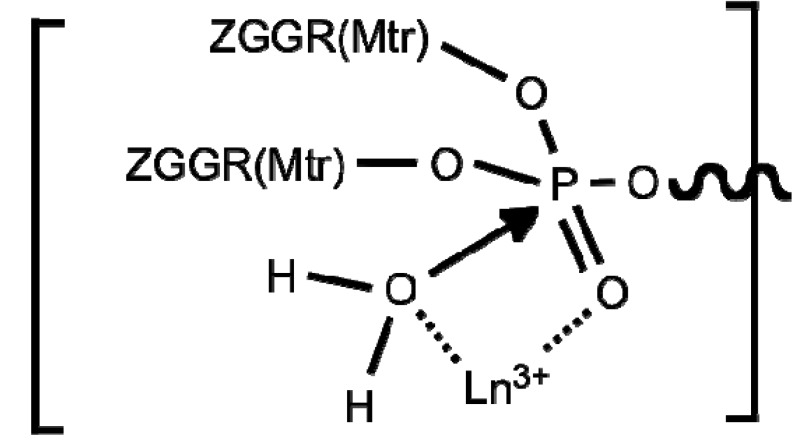
The intermediate during lanthanide-mediated dephosphorylation, which results in cleavage of the two peptide-phosphate bonds and the resin-phosphate bond.

The peptide was cleaved from 100 mg of phosphorylated Wang resin **3** using 50 mM EuCl_3_·6H_2_O in 1.5 mL of water. This Eu(III) solution was first brought to neutral pH by titrating with 1 N NaOH before it was added to the resin. The cleavage solution was incubated for 2 h at r.t. The resin was precipitated with centrifugation and the supernatant was filtered to remove any remaining undisolved solid. This procedure was conducted a second time to cleave any remaining peptides on resin-bound phosphodiesters. The resulting solutions from the two cleavage reactions were combined. The pH was adjusted to 10 to form lanthanide-hydroxide precipitates that were subsequently removed with centrifugation and filtration of the supernatant with a membrane filter. The solution was neutralized with 0.1 N HCl and lyophilized to produce a white powder. The yield was determined by weighing the powder immediately after lyophilization. The total yield of both cleavages indicated that two peptides were synthesized per resin-bound phosphate, which doubled the synthesis scale per phosphate (Figure 3). 

This doubling of the synthesis scale validated our hypothesis that the phosphate group could carry two peptides during SPPS, as shown in Scheme 1 and Figure 3. The yield was 98.8 ± 2.2% for Eu(III)-mediated cleavage relative to the amount of phosphodiester groups on the phosphorylated Wang resin (Table 2). This quantitative yield demonstrates the utility of this method.

**Table 2:** The peptide recovery after treatments with Eu(III) in water or 5% water in TFA.

Reactant	Reactant (μmol)	Cleavage Method	Peptide Product (μmol)	% Yield ^1^
resin-bound phosphate	65.2	first Eu(III)	51.6	79.1%
		second Eu(III)	15.0	23.0%
		sum of 2 Eu(III) cleavages	66.6	102.1%^a^
	99.2	first Eu(III)	71.2	71.8%
		second Eu(III)	25.7	25.9%
		sum of 2 Eu(III) cleavages	96.9	97.7%
average of two trials of resin-bound phosphate	82.2 ± 17	sum of 2 Eu(III) cleavages	81.8 ± 15.1	98.9 ± 1.2%
resin-bound hydroxyl groups	77.0	5% water in TFA	55.2	71.7%
	60.0	5% water in TFA	58.4	97.3%
average of two trials of resin-bound hydroxyl groups	68.5 ± 8.5	5% water in TFA	56.8 ± 1.6	84.5 ± 12.8%

^1^ The crude yield is based on total crude mass.

Free Eu(III) ions were removed from the final peptide product by elevating the pH to form Eu(III)-hydroxide precipitates. The product was analyzed with an arsenazo(III) color test, which showed no evidence of free Eu(III) ions, at least to the detection threshold of ~20 μM [27]. Although trace contents of lanthanide ions below this concentration threshold were acceptable for this report, additional methods that remove traces of lanthanide ions may be needed to further purify similar peptide syntheses, especially for biological applications [28,29,30,31]. The final product also contained phosphate ions. No attempts were made to remove these phosphates, because peptides are often prepared in phosphate-containing buffers during biological applications. The final product did not contain any other cleavage products that may degrade the peptide, which is an improvement relative to other peptide-resin cleavage methods that create free radicals that must be rapidly scavenged [32]. Figure 2A shows that a higher yield would be expected after one cleavage reaction in a mixture of water and EtOH. However, we chose to use only water during peptide cleavage to demonstrate that this protocol has the advantage of being a “green chemistry” method that does not require organic solvents. In addition, this lanthanide-mediated dephosphorylation occurs at neutral pH and produces only phosphate ions as a byproduct, which aids in creating a “green chemistry” method. 

**Figure 4:**
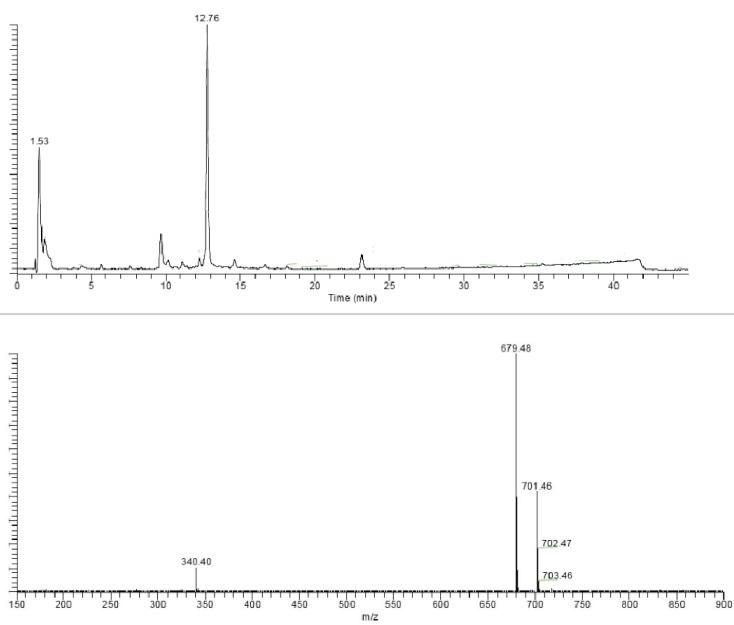
LC-MS results for the peptide cleavage after Eu treatment of the resin.

The final product was analyzed with LC-MS (Figure 4). Each LC-MS analysis showed a single major product with an elution time of 12.76–12.79 min, and a molecular weight of 679.42–679.51 g/mol/charge (calcd. 679.21 g/mol/charge). The MS analysis also showed evidence for a peptide-sodium salt complex, which is commonly observed in Electrospray Ionization MS. These results confirmed that the final peptide product was still protected with CBZ at the amino terminus and with Mtr at the arginine side chain. Therefore, the lanthanide-mediated hydrolysis of the phosphate linker did not affect other peptide protecting groups. The MS also showed that the carboxylate terminus of the peptide was not linked to a phosphate group, which showed that the lanthanide-mediated dephosphorylation efficiently cleaved the carboxylate-phosphate bond either before or after the phosphate was cleaved from the Wang resin (Figure 3). The total purity of the crude product was determined to be an average of 71.8% from LC-MS. The synthesis of a model peptide sequence was sufficient to demonstrate the utility of lanthanide-mediated peptide cleavage during SPPS. Additional studies are warranted to test the synthesis of other peptide sequences using this SPPS methodology, especially for longer sequences, sequences that may aggregate when bound to the phosphorylated Wang resin, and sequences that weakly bind to lanthanide ions.

During this SPPS, peptides were also synthesized onto the unphosphorylated hydroxyl groups on the resin. To assess the SPPS with the unphosphorylated hydroxyl groups, the peptide-resin that was treated with Eu(III) was subsequently treated with 1.5 mL 5% water in TFA to cleave any remaining resin-bound peptides from the resin. After TFA removal, the solids were dispersed in diethyl ether. The diethyl ether layer was removed by centrifugation and the precipitates that remained after removing the diethyl ether were dried *in vacuo*. These precipitates contained the peptides. This entire process was conducted a second time to cleave peptides from another 100 mg of resin **3** to investigate the precision of this method. The peptide products from Eu(III)-mediated cleavage were analyzed with LC-MS. The TFA cleavage produced the peptide with a 84.5 ± 12.8% yield relative to the amount of unphosphorylated hydroxyl groups on the resin (Table 2). This retention of some peptide product on the resin following lanthanide-mediated cleavage is a potential disadvantage, and additional studies are warranted to improve the phosphorylation of the resin to improve SPPS scales. However, this hydroxyl-bound peptide retention following lanthanide-mediated cleavage may be an advantage, as the phosphate-bound peptide product can be selectively cleaved from the phosphorylated resin while leaving other peptides bound to the resin for additional SPPS.

## 3. Experimental

### 3.1. General Procedures

All chemicals were reagent grade commercial samples and were used without further purification. The loading efficiency of phosphate groups on the resin was analyzed quantitatively with a UV/Vis spectrometer (model DU640B, Beckman, Brea, CA, USA). The pH values of solutions in water were measured with a benchtop pH meter (FE20 Five-Easy pH meter, Mettler Toledo, Inc., Columbus, OH, USA). To characterize purity and yield, and to confirm the identity of the final peptide product, the final product was analyzed with a LC-MS system (Finnigan TSQ Quantum Ultra system with a Surveyor autosampler, Surveyor MS pump and Surveyor PDA detector, Thermo Electron Corp., Madison, WI, USA). 

### 3.2. Synthesis of Phosphate-Loaded Resins

Five g of Wang resin **1** (1.2 mmol of hydroxyl groups per gram of resin) was dispersed in trimethylphosphate and stirred for 1 h. Two eq. of phosphorous oxychloride (12 mmol) [33,34] were added and the mixture was heated to 60 °C for 12 h. The suspension was poured into water and stirred for 2 h. The resin was dried in vacuo after washing with water, EtOH, acetone and dichloromethane. The resin was phosphorylated using pyrophosphoric acid and acetylphosphate ester following previous reports [21,35]. The yields of each phosphorylation of **2** are shown in Table 1.

### 3.3. Quantification of the Phosphorylation of a Resin

The phosphate group on the resin **2** was quantified using a Mo-blue assay [22]. To perform this assay, a small amount of resin (~20 mg) was washed three times with EtOH (1.5 mL) and three times with water (1.5 mL). The resin was mixed with 2 N HCl (300 μL) and heated to 95 °C for 30 min. The solution was mixed with Mo-blue assay solution (700 μL) that consisted of 0.82% (NH_4_)_6_Mo_7_O_24_·4H_2_O (300 μL), 2 N H_2_SO_4_ (300 μL) and 10% sodium ascorbate (100 μL) solutions. The Mo-blue solution was freshly prepared immediately before the phosphate assay, because the quantification of phosphate was not reproducible when all three solutions were mixed together and stored for two weeks at room temperature. The solution was incubated at 50 °C for 30 min. The aliquot was analyzed with a UV/Vis spectrometer at 825 nm wavelength along with standard KH_2_PO_4_ solutions that ranged from 0 to 11 mM.

### 3.4. Quantification of Phosphate Cleavage

To demonstrate lanthanide-mediated dephosphorylation of the resin **2** under different conditions, 50 mM YbCl_3_ or EuCl_3_ solution (1.5 mL) was added to separate preparations of phosphorylated resin (300 mg). The phosphate cleavage was tested in distilled water, equal volumes of water and EtOH, 20% piperidine in NMP, and 50% TFA in CH_2_Cl_2_. The slurry was mixed at room temperature or at 50 °C. Small (~20 mg) amounts of resin were periodically extracted from the slurry and washed three times with water to remove lanthanide(III) and phosphate ions. The residual phosphate on the resin was quantified using a Mo-blue assay. The residual phosphate contents were traced for two hours. Each dephosphorylation reaction was repeated to demonstrate reproducibility. This procedure was similar to a previously reported method that dephosphorylated peptides using metal ions at 1 mM concentration for 18 h at 25 °C in 50 mM bis-tris propane [5]. Our method uses a higher concentration of metal ions, for a shorter time period and can be performed only with water.

### 3.5. Solid Phase Peptide Synthesis

Two preparations of phosphate loaded resin **2** (with PO_4_ loadings of 0.326 and 0.496 mmol per gram of resin, respectively) were used for SPPS following Boc-chemistry methods. Firstly, Boc-Arg(Mtr)-OH (4 equiv.) was mixed with HBTU (4 equiv.), HOBt (1 equiv.) and DIEA (6 equiv.) in NMP (5 mL). The coupling reaction was carried out overnight and then the resin was treated with the same reaction mixture for 12 h to perform a double coupling SPPS reaction. The Boc protecting group was removed by treating the resin with 50% TFA in CH_2_Cl_2_ for 30 min. The resin was washed and tested with Kaiser’s color test [36,37]. The resin showed a black color and the solution showed a dark purplish color from this test, which qualitatively demonstrated that the Boc protecting group was removed. The mixture of Boc-Gly-OH (4 equiv.), HBTU (4 equiv.), HOBt (1 equiv.) and DIEA (6 equiv.) in NMP (5 mL) was reacted with the resin for 1 h. The same coupling was carried out a second time to perform a double coupling SPPS reaction. The Boc group was removed with 50% TFA in CH_2_Cl_2_ and small amounts of resin were tested with Kaiser’s color test after washing with CH_2_Cl_2_, acetone, EtOH and ether. Finally, CBZ-Gly-OH (4 equiv.), HBTU (4 equiv.), HOBt (1 equiv.) and DIEA (6 equiv.) in NMP (5 mL) were treated with resin for 1 h. The same reaction was repeated a second time to perform a double coupling SPPS reaction. The resin **3** was washed with NMP, EtOH, CH_2_Cl_2_, acetone and ether and dried *in vacuo*.

### 3.6. Lanthanide-Mediated Peptide Cleavage

After SPPS, the peptide product was cleaved from the phosphodiesters of resin **3** (100 mg) using 50 mM EuCl_3_ in water (1.5 mL) at pH 6.2. The cleavage solution was incubated for 2 h at r.t. The resin was precipitated with centrifugation and the supernatant was filtered to remove any remaining undissolved solids. This procedure was conducted a second time to cleave any remaining peptides on the resin-bound phosphodiesters. The resulting solutions from the two cleavage reactions were combined. The pH was adjusted to 10 with 1 N NaOH to form lanthanide-hydroxide precipitates that were subsequently removed with centrifugation and filtration of the supernatant with a 0.2 μm PVDF membrane filter. The solution was neutralized with 0.1 N HCl solution and lyophilized to produce a white powder. The lanthanide-treated resins were subsequently treated with 5% water (1.5 mL) in TFA to cleave any remaining peptides from the resin. After TFA removal, the solids were dispersed in diethyl ether. The ether layer was removed by centrifugation and the precipitates were dried *in vacuo*. This entire process was conducted a second time to cleave peptides from another 100 mg of resin **3** to investigate the precision of this method. The peptide products from Eu(III)-mediated cleavage were analyzed with LC-MS. The LC used a 150 × 2.1 mm size, 5 μm particle size BDS Hypersil C-18 column (Thermo Scientific, Inc., Waltham, MA, USA). The eluting solvent consisted of a mixture of acetonitrile and 0.01% TFA, which was ramped from 10% acetonitrile to 70% acetonitrile over 40 min at a flow rate of 0.3 mL/min. The MS used electrospray ionization in the positive ionization mode, with a TSQ Quantum detector (Thermo Electron, Inc.) that scanned from a molecular weight of 150 to 900 amu.

## 4. Conclusions

These results demonstrated lanthanide-mediated dephosphorylation on a polymeric support, which was carried out in neutral aqueous media. The rate of dephosphorylation was accelerated by an increase in temperature or the addition of ethanol, which presumably improved resin swelling. The phosphorylated resin was used to synthesize a peptide following a conventional Boc-SPPS protocol. The peptide, CBZ-Gly-Gly-Arg(Mtr)-OH, was cleaved from the resin by lanthanide-mediated dephosphorylation in neutral aqueous media. The protecting groups of N-terminus and arginine side chain were retained after the cleavage step, which indicates that this orthogonal cleavage method can expand the utility of SPPS for bioconjugate chemistry applications. 
